# Coupled Relational Symbolic Execution for Differential Privacy

**DOI:** 10.1007/978-3-030-72019-3_8

**Published:** 2021-03-23

**Authors:** Gian Pietro Farina, Stephen Chong, Marco Gaboardi

**Affiliations:** Imperial College, London, UK; 9 University at Buffalo SUNY, Buffalo, USA; 10 Harvard University, Cambridge, USA; 11 Boston University, Boston, USA

## Abstract

Differential privacy is a de facto standard in data privacy with applications in the private and public sectors. Most of the techniques that achieve differential privacy are based on a judicious use of randomness. However, reasoning about randomized programs is difficult and error prone. For this reason, several techniques have been recently proposed to support designer in proving programs differentially private or in finding violations to it.

In this work we propose a technique based on symbolic execution for reasoning about differential privacy. Symbolic execution is a classic technique used for testing, counterexample generation and to prove absence of bugs. Here we use symbolic execution to support these tasks specifically for differential privacy. To achieve this goal, we design a relational symbolic execution technique which supports reasoning about probabilistic coupling, a formal notion that has been shown useful to structure proofs of differential privacy. We show how our technique can be used to both verify and find violations to differential privacy.

### 1 Introduction

Differential Privacy [8] has become a de facto gold standard definition of privacy for statistical analysis. This success is mostly due to the generality of the definition, its robustness and compositionality. However, getting differential privacy right in practice is a hard task. Even privacy experts have released fragile code subject to attacks [13, 17] and published incorrect algorithms [16]. This challenge has motivated the development of techniques to support programmers to show their algorithms differentially private. Among the techniques that have been proposed there are type systems [12,18,20,24,26], methods based on model checking and program analysis [2,15,22,23], and program logics [3,4,21]. Several works have also focused on developing techniques to find violations to differential privacy [2, 5, 6, 23, 27]. Most of these works focus only on either verifying a program differentially private or finding violations. Exceptions are the recent works by Barthe et al. [2] and Wang et al. [23] (developed concurrently to our work) which propose method that can instead address both.

Motivated by this picture, we propose a new technique named Coupled Relational Symbolic Execution (CRSE), which supports proving and finding violation to differential privacy. Our technique is based on two essential ingredients: relational symbolic execution [10] and approximate probabilistic couplings [3].

**Relational Symbolic Execution.** Symbolic execution is a classic technique used for bug finding, testing and proving. In symbolic execution an evaluator executes the program which consumes symbolic inputs instead of concrete ones. The evaluator follows, potentially, all the execution paths the program could take and collects constraints over the symbolic values, corresponding to these paths. Similarly, in relational symbolic execution [10] (RSE) one is concerned with bug finding, testing, or proving for *relational properties*. These are properties about two executions of two potentially different programs. RSE executes two potentially different programs in a symbolic fashion and exploits relational assumptions about the inputs or the programs in order to reduce the number of states to analyze. This is effective when the codes of the two programs share some similarities, and when the property under consideration is relational in nature, as in the case of differential privacy.

**Approximate Probabilistic Couplings.** Probabilistic coupling is a proof technique useful to lift a relation over the support of a joint distribution to a relation over the two probability marginals of the joint. This allows one to reason about relations between probability distributions by reasoning about relations on their support, which can be usually done in a symbolic way. In this approach the actual probabilistic reasoning is confined to the soundness of the verification system, rather than being spread everywhere in the verification effort. A relaxation of the notion of coupling, called *approximate probabilistic coupling* [3, 4], has been designed to reason about differential privacy. This can be seen as a regular probabilistic coupling with some additional parameters describing how close the two probability distribution are.

In this work, we combine these two approaches in a framework called Coupled Relational Symbolic Execution. In this framework, a program is executed in a relational and symbolic way. When some probabilistic primitive is executed, CRSE introduces constraints corresponding to the existence of an approximate probabilistic coupling on the output. These constraints are combined with the constraints on the execution traces generated by symbolically and relationally executing other non-probabilistic commands. These combined constraints can be exploited to reduce the number of states to analyze. When the execution is concluded CRSE checks whether there is a coupling between the two outputs, or whether there is some violation to the coupling. We show the soundness of this approach for both proving and refuting differential privacy. However, for finding violations, one cannot reason only symbolically, and since checking a coupling directly can be computationally expensive, we devise several heuristics which can be used to facilitate this task. Using these techniques, CRSE allows one to verify differential privacy for an interesting class of programs, including programs working on countable input and output domains, and to find violations to programs that are not differentially private.

CRSE is not a replacement for other techniques that have been proposed for the same task, it should be seen as an additional method to put in the set of tools of the privacy developer which provides a high level of generality. Indeed, by being a totally symbolic technique, it can leverage a plethora of current technologies such as SMT solvers, algebraic solvers, and numeric solvers.

Summarizing, the contribution of our work are:

- We combine relational symbolic execution and approximate probabilistic coupling in a new technique: Coupled Relational Symbolic Execution.
- We show CRSE sound for proving programs differentially private
- We devise a set of heuristic - one of them sound, and the others useful - that can help a programmer in finding violations to differential privacy.
- We show how CRSE can help in proving and refuting differential privacy for an interesting class of programs

Most of the proofs are omitted here, more details can be found in [9,11].

### 2 CRSE Informally

We will introduce CRSE through three examples of programs showing potential errors in implementations of differentially private algorithms. Informally, a randomized function $A$ over a set of databases $\mathcal{D}$ is $\epsilon$-differential privacy ($\epsilon$-DP) if it maps two databases $D_1$ and $D_2$ that differ for the data of one single individual (denoted $D_1 \sim D_2$) to output distributions that are indistinguishable up to some value $\epsilon$ - usually referred to as the privacy budget. This is formalized by requiring that for every $D_1 \sim D_2$ and for every $u$: $\Pr[\mathcal{A}(D_1) = u] \leq e^{\epsilon} \Pr[\mathcal{A}(D_2) = u]$. The smaller the $\epsilon$, the more privacy is guaranteed.

**Algorithm 1**
A bad use of Randomized Response

**Input:** $\epsilon \in \mathcal{R}^+, x_1, x_2 \in \{true, false\}$
**Precondtion:** $x_1 \neq x_2$
**Postcondition:** $o_1 = o_2 \wedge \epsilon_c \leq \epsilon$

```
1: o ← RR_ε(x)
2: return o
```

Fig. 1: Algorithm 1 is not $\epsilon$-DP.

*Randomized response with wrong noise.* A standard primitive to achieve differential privacy when the data is a single boolean is randomized response [25]. We will use this (simplified) primitive to give an idea of how CRSE works. This primitive can be actually reduced to the primitives that CRSE uses and so it won't be included in later sections. The primitive $RR_p(b)$ takes in input $p \in (\frac{1}{2}, 1)$ and a boolean $b$ and it outputs $b$ with probability $p$, and $\bar{b}$ with probability $1 - p$. By unfolding the definition of differential privacy it is easy to see that this primitive is $log[-p/(p-1)]$-DP. This is internalized in CRSE thanks to the the existence of an $log[-p/(p-1)]$-approximate lifting (Definition 2) of the equality relation $=$ between the distributions $RR_p(b)$ and $RR_p(\bar{b})$. When CRSE executes line 1, it assumes that $o_1 = o_2$ and it sets a counter $\epsilon_c$, representing the privacy budget required by the primitive, to $\log[-\frac{\epsilon}{\epsilon-1}]$. In order to check whether this program is actually $\epsilon$-DP it will then try to check whether this set of conditions implies the postcondition $\Psi \equiv o_1 = o_2 \wedge \epsilon_c \leq \epsilon$. This implication will fail. Indeed, there are value of $\epsilon$, say $\epsilon = 0.7$, which give a value of $\epsilon_c$ which is actually greater than $\epsilon$. This shows that the user may have confused the $\epsilon$ parameter with the parameter $p$ that the randomized response primitive takes in input. If the user substituted line 1 with the following $p \leftarrow \frac{e^\epsilon}{1+e^\epsilon}; o \xleftarrow{\$} RR_p(x)$, then CRSE would have considered the following conditions instead: $o_1 = o_2$ and $\epsilon_c = \log[-\frac{p}{p-1}] \wedge p = \frac{e^\epsilon}{1+e^\epsilon}$. These conditions would then imply the postcondition $\Psi$ proving the correcteness of the program.

The intuition behind this proof is that everytime CRSE executes a random assignment of the form $o \xleftarrow{\$} RR_p(x)$, it is allowed to assume that $o_1 = o_2$ as long as it spends a certain amount of privacy budget, i.e. $log[-\frac{p}{p-1}]$. These assumptions are recorded in a set of constraints which is then used to see if it implies the condition that two output variables are equal and the budget spent does not exceed $\epsilon$. As a consequence of the definition of approximate lifting, this implies differential privacy (Lemma 2). If this fails, CRSE will provide a counterexample in the form of values for the inputs $x_1, x_2, \epsilon, p$. Such counterexamples to the postcondition do not necessarily denote a counterxampled to the privacy of the algorithm (as we will see later the logic of couplings which CRSE is based on is not complete w.r.t the differential privacy notion) but only potential candidates, and hence need to be further checked.

**Algorithm 2** A buggy Above Threshold

**Input:** $t, \epsilon \in \mathbb{R}, D \in \mathcal{D}, q[i] : \mathcal{D} \to \mathbb{N}$
**Output:** $o : [\perp^i, z, \perp^{n-i-1}]$
**Precondition:** $D_1 \sim D_2 \Rightarrow |q[i](D_1) - q[i](D_2)| \leq 1$
**Postcondition:** $o_1 = o_2 \wedge \epsilon_c \leq \epsilon$

$o \leftarrow \perp^n; r \leftarrow n+1$
$\hat{t} \xleftarrow{\$} lap_{\frac{\epsilon}{2}}(t)$
**for** $(i$ **in** $1{:}n)$ **do**
  $\hat{s} \xleftarrow{\$} lap_{\frac{\epsilon}{4}}(q[i](D))$
  **if** $\hat{s} > \hat{t} \wedge r = n+1$ **then**
    $o[i] \leftarrow \hat{s}; r \leftarrow i$
**return** $o$

**Algorithm 3** Another buggy Above Threshold

**Input:** $t, \epsilon \in \mathbb{R}, D \in \mathcal{D}, q[i] : \mathcal{D} \to \mathbb{N}$
**Output:** $o \in \{\perp, \top\}^n$
**Precondition:** $D_1 \sim D_2 \Rightarrow |q[i](D_1) - q[i](D_2)| \leq 1$
**Postcondition:** $o_1 = o_2 \wedge \epsilon_c \leq \epsilon$

$\hat{t} \xleftarrow{\$} lap_{\frac{\epsilon}{2}}(t)$
**for** $(i$ **in** $1{:}n)$ **do**
  **if** $q[i](D) \geq \hat{t}$ **then**
    $o[i] \leftarrow \top$
  **else**
    $o[i] \leftarrow \perp$
**return** $o$

*Two buggy Sparse Vector implementations.* The next two examples are variations of the algorithm *above threshold*, a component of the *sparse vector* technique, a classical technique which is still subject of studies for improvement [7,14]. Given a numeric threshold, an array of numeric queries of length $n$, and a dataset, this algorithm returns the index of the first query whose result exceeds the threshold - and potentially it should also return the value of that query. This should be done in a way that preserves differential privacy. To do this in the right way, a program should add noise to the threshold, even if it is not sensitive data, add noise to each query, compare the values, and return the index of the first query for which this comparison succeed. The noise that is usually added is sampled from the Laplace distribution, one of the main primitive in differential privacy. The analysis of this algorithm is rather complex: it uses the noise on the threshold as a way to pay only once for all the queries that are below the threshold, and the noise on the queries to pay for the first and only query that is above the threshold, if any. Due to this complex analysis [16], this algorithm has been a benchmark for tools for reasoning about differential privacy [2,3,26].

Algorithm 2 has a bug making the (whole) program not differentially private, for values of $n$ greater than 4. The program initializes an array of outputs $o$ to all bottom values, and a variable $r$ to $n+1$ which will be used as guard in the main loop. It then adds noise to the threshold, and iterates over all the queries adding noise to their results. If one of the noised-results is above the noisy threshold it saves the value in the array of outputs and updates the value of the guard variable, causing it to exit the main loop. Otherwise it keeps iterating. The bug is returning the value of the noisy query that is above the threshold and not only its index, as done by the instruction in red in line 6 - this is indeed not enough for guaranteeing differential privacy. For $n < 5$ this program can be shown $\epsilon$-differentially private by using the composition property of differential privacy that says that the k-fold composition of $\epsilon$-DP programs is $k\epsilon$-differentially private (Section 3). However, for $n \geq 5$ the more sophisticated analysis we described above fails. The proof principle CRSE will use to try to show this program $\epsilon$-differentially private is to prove the assertion $o_1 = \iota \implies o_2 = \iota \wedge \epsilon_c \leq \epsilon$, for every $\iota \leq n$ - the soundness of this principle has been proved in [3]. That is, CRSE will try to prove the following assertions (which would prove the program $\epsilon$-differentially private):

- $o_1 = [\hat{s}_1, \bot, \dots, \bot] \implies o_2 = [\hat{s}_1, \bot, \dots, \bot] \wedge \epsilon_c \leq \epsilon$
- $o_1 = [\bot, \hat{s}_1, \dots, \bot] \implies o_2 = [\bot, \hat{s}_1, \dots, \bot] \wedge \epsilon_c \leq \epsilon$

...
- $o_1 = [\bot, \dots, \hat{s}_1] \implies o_2 = [\bot, \dots, \hat{s}_1] \wedge \epsilon_c \leq \epsilon$

While proving the first assertion, CRSE will first couple at line 3 the threshold as $\hat{t}_1 + k_0 = \hat{t}_2$, for $k_0 > 1$ where 1 is the sensitivity of the queries, which is needed to guarantee that all the query results below the threshold in one run stay below the threshold in the other run, then, it will increase appropriately the privacy budget by $k_0\frac{\epsilon}{2}$. As a second step it will couple $\hat{s}_1 + k_1 = \hat{s}_2$ in line 4. Now, the only way for the assertion $o_1 = [\hat{s}_1, \bot, \bot] \implies o_2 = [\hat{s}_1, \bot, \bot]$ to hold, is guaranteeing that both $\hat{s_1} = \hat{s}_2$ and $\hat{s}_1 \geq t_1 \implies \hat{s_2} \geq t_2$ hold. But these two assertions are not consistent with each other because $k_0 \geq 1$. That is, the only way, using these coupling rules, to guarantee that the run on the right follows the same branches of the run on the left (this being necessary for proving the postcondition) is to couple the samples $\hat{s}_1$ and $\hat{s}_2$ so that they are different, this necessarily implying the negation of the postcondition. This would not be the case if we were returning only the index of the query, since we can have that both the queries are above the threshold but return different values. Indeed, by substituting line 7 with $o[i] \xleftarrow{\$} \top$ the program can be proven $\epsilon$-differentially private. So the *refuting* principle CRSE will use here is the one that finds a trace on the left run such that the only way the right run can be forced to follow it is by making the output variables different.

A second example with bug of the above threshold algorithm is shown in Figure 3. In this example, in the body of the loop, the test is performed between the noisy threshold and the actual value of the query on the database - that is, we don't add noise to the query. CRSE will use for this example another *refuting* principle based on reachability. In particular, it will vacuously couple the two thresholds at line 1. That is it will not introduce any relation between $\hat{t}_1$, and $\hat{t}_2$. CRSE will then search for a trace which is satisfiable in the first run but not in the second one. This translates in an output event which has positive probability on the first run but 0 probability in the second one leading to an unbounded privacy loss, and making the algorithm not $\epsilon$-differentially private for all finite $\epsilon$. Interestingly this unbounded privacy loss can be achieved with just 2 iterations.

### 3 Preliminaries

Let $A$ be a denumerable set, a *subdistribution* over A is a function $\mu : A \to [0,1]$ with weight $\sum_{a \in A} \mu(a)$ less or equal than 1. We denote the set of subdistributions over $A$ as $\mathbf{sdistr}(A)$. When a subdistribution has weight equal to 1, then we call it a *distribution*. We denote the set of distributions over $A$ by $\mathbf{distr}(A)$. The *null* subdistribution $\mu_0 : A \to [0,1]$ assigns to every element of $A$ mass 0. The Dirac's distribution $\mathbf{unit}(a) : A \to [0,1]$, defined for $a \in A$ as $\mathbf{unit}(a)(x) \equiv 1$ if $x = a$, and $\mathbf{unit}(a)(x) \equiv 0$, otherwise. The set of subprobability distributions can be given the structure of a *monad*, with unit the function **unit**. We have also a function $\mathbf{bind} \equiv \lambda\mu.\lambda f.\lambda a. \sum_{b \in \mathcal{O}'} \mu(b) \cdot f(b)(a)$ allowing us to compose subdistributions (as we compose monads). We will use the notion of $\epsilon$-divergence $\Delta_\epsilon(\mu_1, \mu_2)$ between two subdistributions $\mu_1, \mu_2 \in \mathbf{sdistr}(A)$ to define approximate coupling, this is defined as: $\Delta_\epsilon(\mu_1, \mu_2) \equiv \sup_{E \subseteq O} \big(\mu_1(E) - \exp(\epsilon) \cdot \mu_2(E)\big)$.

Formally, differential privacy is a property of a probabilistic program:

**Definition 1 (Differential Privacy [8]).** *Let $\epsilon \geq 0$ and $\sim \subseteq \mathcal{D} \times \mathcal{D}$. A program $\mathcal{A} : \mathcal{D} \to \mathbf{distr}(\mathcal{O})$ is $\epsilon$-differentially private with respect to $\sim$ iif $\forall D \sim D'. \forall u \in \mathcal{O}$:*

$$\Pr[\mathcal{A}(D) = u] \leq e^{\epsilon} \Pr[\mathcal{A}(D') = u]$$

The adjacency relation $\sim$ over the set of databases $\mathcal{D}$ models which pairs of input databases should be indistinguishable to an adversary. In its most classical definition, $\sim$ relates databases that differ in one record in terms of hamming distance. Differentially private programs can be composed [8]: given programs $A_1$ and $A_2$, respectively $\epsilon_1$ and $\epsilon_2$ differentially private, their sequential composition $A(D) \equiv A_2(\langle A_1(D), D\rangle)$ is $\epsilon_1 + \epsilon_2$-differentially private. We say that a function $f : \mathcal{D} \to \mathbb{Z}$ is $k$ *sensitive* if $|f(x) - f(y)| \leq k$, for all $x \sim y$. Functions with bounded sensitivity can be made differentially private by adding Laplace noise:

**Lemma 1 (Laplace Mechanism [8]).** *Let $\epsilon > 0$, and assume that $f : \mathcal{D} \mapsto \mathbb{Z}$ is a $k$ sensitive function with respect to $\sim \subseteq \mathcal{D} \times \mathcal{D}$. Then the randomized algorithm mapping $d$ to $f(D) + \nu$, where $\nu$ is sampled from a discrete version of the Laplace distribution with scale $\frac{1}{\epsilon}$, is $k\epsilon$-differentially private w.r.t to $\sim$.*

The notion of approximate probabilistic coupling is internalized by the notion of approximate lifting [3].

**Definition 2.** *Given $\mu_1 \in \mathbf{distr}(A), \mu_2 \in \mathbf{distr}(B)$, a relation $\Psi \subseteq A \times B$, and $\epsilon \in \mathbb{R}$, we say that $\mu_1, \mu_2$ are related by the $\epsilon$ approximate lifting of $\Psi$, denoted $\mu_1(\Psi)^\epsilon \mu_2$, iff there exists $\mu_L, \mu_R \in \mathbf{distr}(A \times B)$ such that: 1) $\lambda a. \sum_b \mu_L(a, b) = \mu_1$ and $\lambda b. \sum_a \mu_R(a, b) = \mu_2$, 2) $\{(a, b) | \mu_L(a, b) > 0 \vee \mu_R(a, b) > 0\} \subseteq \Psi$, 3) $\Delta_\epsilon(\mu_L, \mu_R) \leq 0$.*

Approximate lifting satisfies the following fundamental property [3]:

**Lemma 2.** *Let $\mu_1, \mu_2 \in \mathbf{distr}(A), \epsilon \geq 0$. Then $\Delta_\epsilon(\mu_1, \mu_2) \leq 0$ iff $\mu_1(=)^\epsilon \mu_2$.*

From Lemma 2 we have that an algorithm $A$ is $\epsilon$-differentially private w.r.t to $\sim$ iff $A(D_1)(=)^\epsilon A(D_2)$ for all $D_1 \sim D_2$. The next lemma [3], finally, casts the Laplace mechanisms in terms of couplings:

**Lemma 3.** *Let $L_{v_1,b}, L_{v_2,b}$ two Laplace random variables with mean $v_1$, and $v_2$ respectively, and scale $b$. Then*

$$L_{v_1,b} \{(z_1, z_2) \mid z_1 + k = z_2 \in \mathbb{Z} \times \mathbb{Z}\}^{|k+v_1-v_2|\epsilon} L_{v_2,b},$$

*for all $k \in \mathbb{Z}, \epsilon \geq 0$.*

### 4 Concrete languages

In this section we sketch the two CRSE concrete languages, the unary one PFOR and the relational one RPFOR. These will be the basis on which we will design our *symbolic* languages in the next section.

#### 4.1 PFOR

PFOR is a basic FOR-like language with arrays, to represent databases and other data structures, and probabilistic sampling from the Laplace distribution. The full syntax is pretty standard and we fully present it in the extended version [11]. In the following we have a simplified syntax:

$$\mathcal{C} \ni c ::= \texttt{skip} \mid c;c \mid x \leftarrow e \mid x \xleftarrow{\$} lap_e(e) \mid \texttt{if } e \texttt{ then } c \texttt{ else } c \mid \ldots$$

The set of commands $\mathcal{C}$ includes assignments, the `skip` command, sequencing, branching, and (not showed) array assignments and looping construct. Finally, we also include a primitive instruction $x \xleftarrow{\$} lap_{e_2}(e_1)$ to model random sampling from the Laplace distribution. Arithmetic expressions $e \in \mathcal{E}$ are built out of integers, array accesses and lengths, and elements in $\mathcal{X}_p$. The set $\mathcal{X}_p$ contains values denoting random expressions, that is values coming from a random assignment or arithmetic expressions involving such values. We will use capital letters such as $X, Y, \ldots$ to range over $\mathcal{X}_p$. The set of values is $\mathcal{V} \equiv \mathbb{Z} \cup \mathcal{X}_p$. In Figure 2, we introduce a grammar of constraints for random expressions, where $X$ ranges over $\mathcal{X}_p$ and $n, n_1, n_2 \in \mathbb{Z}$. The simple constraints in the syntactic categories $ra$ and $re$ record that a random value is either associated with a specific distribution, or that the computation is conditioned on some random expression being greater than 0 or less than or equal than 0. The former constraints, as we will see, come from branching instructions. We treat constraint lists $p, p'$, in Figure 2 as lists of simple constraints and hence, from now on, we will use the infix operators :: and @, respectively, for appending a simple constraint to a constraint and for concatenating two constraints. The symbol [] denotes the empty list of probabilistic constraints. Environments in the set $\mathcal{M}$, or probabilistic memories, map program variables to values in $\mathcal{V}$, and array names to elements in $\mathbf{Array} \equiv \bigcup_i \mathcal{V}^i$, so the type of a memory $m \in \mathcal{M}$ is $\mathbb{V} \to \mathcal{V} \cup \mathbb{A} \to \mathbf{Array}$. We will distinguish between probabilistic concrete memories in $\mathcal{M}$ and concrete memories in the set $\mathcal{M}_{\mathrm{c}} \equiv \mathbb{V} \to \mathbb{Z} \cup \mathbb{A} \to \bigcup_i \mathbb{Z}^i$. Probabilistic concrete memories are meant to denote subdistributions over the set of concrete memories $\mathcal{M}_c$.

$$
\begin{aligned}
ra &::= X \xleftarrow{\$} lap_{n_2}(n_1) \\
re &::= n \mid X \mid re \oplus re \\
P \ni p &::= X = re \mid re > 0 \mid \\
&\quad re \leq 0 \mid ra \mid p :: P \mid []
\end{aligned}
$$

Fig. 2: Probabilistic constraints

Expressions in PFOR are given meaning through a big-step evaluation semantics specified by a judgment of the form: $\langle m, e, p \rangle \downarrow_{\mathrm{c}} \langle v, p' \rangle$, where $m \in \mathcal{M}, e \in \mathcal{E}, p, p' \in P, v \in \mathcal{V}$. The judgments reads as: expression $e$ reduces to the value $v$ and probabilistic constraints $p'$ in an environment $m$ with probabilistic concrete constraints $p$. We omit the rules for this judgment here, but we will present similar rules for the symbolic languages in the next section. Commands are given

$$
\textbf{if-false}\ \frac{\langle m, e, p \rangle \downarrow_{\mathrm{c}} \langle v, p' \rangle \qquad v \in \mathbb{Z} \qquad v \leq 0}{\langle m, \texttt{if}\ e\ \texttt{then}\ c_1\ \texttt{else}\ c_2, p \rangle \to_{\mathrm{c}} \langle m, c_2, p' \rangle}
$$

$$
\textbf{if-true-prob}\ \frac{\langle m, e, p \rangle \downarrow_{\mathrm{c}} \langle v, p' \rangle \qquad v \in \mathcal{X}_p \qquad p'' \equiv p' @ v > 0}{\langle m, \texttt{if}\ e\ \texttt{then}\ c_1\ \texttt{else}\ c_2, p \rangle \to_{\mathrm{c}} \langle m, c_1, p'' \rangle}
$$

$$
\textbf{lap-ass}\ \frac{\begin{array}{c}\langle m, e_1, p \rangle \downarrow_{\mathrm{c}} \langle n_1, p_1 \rangle \qquad \langle m, e_2, p_1 \rangle \downarrow_{\mathrm{c}} \langle n_2, p_2 \rangle \qquad n_2 > 0 \\ X\ \mathbf{fresh}(\mathcal{X}_p) \qquad p' \equiv p_1 @ X = lap_{n_2}(n_1)\end{array}}{\langle m, x \xleftarrow{\$} lap_{e_2}(e_1), p \rangle \to_{\mathrm{c}} \langle m[x \mapsto X], \texttt{skip}, p' \rangle}
$$

Fig. 3: PFOR selected rules meaning through a small-step evaluation semantics specified by a judgment of the form: $\langle m, c, p\rangle \to_{\mathrm{c}} \langle m', c', p'\rangle$, where $m, m' \in \mathcal{M}, c, c' \in \mathcal{C}, p, p' \in P$. The judgment reads as: the probabilistic concrete configuration $\langle m, c, p\rangle$ steps in to the probabilistic concrete configuration $\langle m', c', p'\rangle$. Figure (3) shows a selection of the rules defining this judgment. Most of the rules are self-explanatory so we only describe the ones which are non standard. Rule **lap-ass** handles the random assignment. It evaluates the mean $e_1$ and the scale $e_2$ of the distribution and checks that $e_2$ actually denotes a positive number. The semantic predicate **fresh** asserts that the first argument is drawn nondeterministically from the second argument and that it was never used before in the computation. Notice that if one of these two expressions reduces to a probabilistic symbolic value the computation halts. Rule **if-true-prob** (and **if-false-prob**) reduces the guard of a branching instruction to a value. If the value is a probabilistic symbolic constraint then it will nondeterministically choose one of the two branches recording the choice made in the list of probabilistic constraints. If instead the value of the guard is a numerical constant it will choose the right branch deterministically using the rules **if-false** and **if-true** (not showed).

We call a probabilistic concrete configuration of the form $\langle m, \mathtt{skip}, p\rangle$ final. A set of concrete configurations $\mathscr{D}$ is called final and we denote it by $\mathbf{Final}(\mathscr{D})$ if all its concrete configurations are final. We will use this predicate even for sets of sets of concrete configurations with the obvious lifted meaning. As clear from the rules a run of a PFOR program can generate many different final concrete configurations. A different judgment of the form $\mathscr{D} \Rightarrow_{\mathrm{c}} \mathscr{D}'$, where $\mathscr{D}, \mathscr{D}' \in \mathcal{P}(\mathcal{M} \times \mathcal{C} \times P)$, and in particular its transitive and reflexive closure ( $\Rightarrow_{\mathrm{c}}^{*}$), will help us in collecting all the possible final configurations stemming from a computation. We have only one rule that defines this judgment:

**Sub-distr-step**

$$\frac{\langle m, c, p\rangle \in \mathscr{D} \quad \mathscr{D}' \equiv (\mathscr{D} \setminus \{\langle m, c, p\rangle\}) \cup \{\langle m', c', p'\rangle \mid \langle m, c, p\rangle \to_{\mathrm{c}} \langle m', c', p'\rangle\}}{\mathscr{D} \Rightarrow_{\mathrm{c}} \mathscr{D}'}$$

Rule **Sub-distr-step** nondeterministically selects a configuration $s = \langle m, c, p\rangle$ from $\mathscr{D}$, removes $s$ from it, and adds to $\mathscr{D}'$ all the configurations $s'$ that are reachable from $s$.

In section 3 we defined the notions of lifting, coupling and differential privacy using subdistributions in the form of functions from a set of atomic events to the interval $[0, 1]$. The semantics of the languages proposed so far though only deal with subdistributions represented as set of concrete probabilistic configurations. We now show how to map the latter to the former. In Figure 4 we define a translation function ($[\![\cdot;\cdot]\!]^{\mathbf{mp}}$) and, auxiliary functions as well, between a single probabilistic concrete configuration and a subdistribution defined using the $\mathbf{unit}(\cdot)/\mathbf{bind}(\cdot,\cdot)$ constructs. We make use of the constant subdistribution $\mu_0$ which maps every element to mass 0, and is usually referred to as the *null* subdistribution, also by $lap_{n_2}(n_1)(z)$ we denote the mass of (discrete version of) the Laplace distribution centered in $n_1$ with scale $n_2$ at the point $z$.

The idea of the translation is that we can transform a probabilistic concrete memory $m_s \in \mathcal{M}$ into a distribution over fully concrete memories in $\mathcal{M}_c$ by

$$
\begin{array}{ll}
[\![m_s;p]\!]^{\mathbf{mp}} & = \mathbf{bind}([\![p]\!]^{\mathbf{P}}, (\lambda s_o.\mathbf{unit}(s_o(m_s)))) \\
[\![[]]\!]^{\mathbf{P}} & = \mathbf{unit}([]) \\
[\![X = re :: p']\!]^{\mathbf{P}} & = \mathbf{bind}([\![p']\!]^{\mathbf{P}}, \lambda s_o.\mathbf{bind}([\![re]\!]^{\mathbf{re}}_{s_o}, \lambda z_o.\mathbf{unit}(X = z_o :: s_o))) \\
[\![re > 0 :: p']\!]^{\mathbf{P}} & = \mathbf{bind}([\![p']\!]^{\mathbf{P}}, \lambda s_o.\mathbf{bind}([\![re]\!]^{\mathbf{re}}_{s_o}, \lambda z_o.\text{if } (z_o > 0) \text{ then } \mathbf{unit}(z_o) \text{ else } \mu_0)) \\
[\![re \leq 0 :: p']\!]^{\mathbf{P}} & = \mathbf{bind}([\![p']\!]^{\mathbf{P}}, \lambda s_o.\mathbf{bind}([\![re]\!]^{\mathbf{re}}_{s_o}, \lambda z_o.\text{if } (z_o \leq 0) \text{ then } \mathbf{unit}(z_o) \text{ else } \mu_0)) \\
[\![lap_{n_2}(n_1)]\!]^{\mathbf{re}}_s & = \lambda z.lap_{n_2}(n_1)(z) \\
[\![n]\!]^{\mathbf{re}}_s & = \mathbf{unit}(n) \\
[\![X]\!]^{\mathbf{re}}_s & = \mathbf{unit}(s(X)) \\
[\![re_1 \oplus re_2]\!]^{\mathbf{re}}_s & = \mathbf{bind}([\![re_1]\!]^{\mathbf{re}}_s, \lambda v_1.\mathbf{bind}([\![re_2]\!]^{\mathbf{re}}_s, \lambda v_2.\mathbf{unit}(v_1 \oplus v_2)))
\end{array}
$$

Fig. 4: Translation from configurations to subdistributions.

sampling from the distributions of the probabilistic variables defined in $m_s$ in the order they were declared which is specified by the probabilistic path constraints. To do this we first build a substitution for the probabilistic variable which maps them into integers and then we perform the substitution on $m_s$. Given a set of probabilistic concrete memories we can then turn them in a subdistribution by summing up all the translations of the single probabilistic configurations. Indeed, given two subdistributions $\mu_1, \mu_2$ defined over the same set we can always define the subdistribution $\mu_1 + \mu_2$ by the mapping $(\mu_1 + \mu_2)(a) = \mu_1(a) + \mu_2(a)$.

The following Lemma states an equivalence between these two representations of probability subdistributions. The hypothesis of the theorem involve a well-formedness judgment, $m \vdash p$, which has not been specified for lack of space but can be found in the extended version [11], it deals with well-formedness of the probabilistic path constraint $p$ with respect to the concrete probabilistic memory $m$.

**Lemma 4.** *If* $m \vdash p$ *and* $\{\langle m, c, p\rangle\} \Rightarrow^*_{\mathrm{c}} \{\langle m_1, \mathtt{skip}, p_1\rangle, \ldots, \langle m_n, \mathtt{skip}, p_n\rangle\}$ *then* $\mathbf{bind}([\![m;p]\!]^{\boldsymbol{mp}}, [\![c]\!]_{\mathcal{C}}) = \sum_{i=1}^{n} [\![m_i;p_i]\!]^{\boldsymbol{mp}}$

This lemma justifies the following definition for the semantics of a program.

**Definition 3.** *The semantics of a program* $c$ *executed on memory* $m$ *and probability path constraint* $p_0$ *is* $[\![c]\!]_{\mathcal{C}}(m_0, p_0) \equiv \sum_{(m,\mathtt{skip},p)\in\mathscr{D}} [\![m;p]\!]^{\boldsymbol{mp}}$,

*when* $\{\langle m, c, p\rangle\} \Rightarrow^*_{\mathrm{c}} \mathscr{D}$, ***Final***$(\mathscr{D})$, *and* $m_0 \vdash p_0$. *If* $p_0 = []$ *we write* $[\![c]\!]_{\mathcal{C}}(m_0)$.

### 4.2 RPFOR

In order to be able to reason about differential privacy we will build on top of PFOR a relational language called RPFOR with a relational semantics dealing with pair of traces. Intuitively, an execution of a single RPFOR program represents the execution of two PFOR programs. Inspired by the approach of [19], we extend the grammar of PFOR with a pair constructor $\langle\cdot|\cdot\rangle$ which can be used at the level of values $\langle v_1|v_2\rangle$, expressions $\langle e_1|e_2\rangle$, or commands $\langle c_1|c_2\rangle$, where $c_i, e_i, v_i$ for $i \in \{1, 2\}$ are commands, expressions, and values in PFOR. This entails that pairs cannot be nested. This syntactic invariant is preserved by the rules handling the branching instruction. Pair constructs are used to indicate where commands, values, or expressions might be different in the two unary executions represented by a single RPFOR execution. The set of expressions and commands in RPFOR, $\mathcal{E}_{\mathrm{r}}, \mathcal{C}_{\mathrm{r}}$ are generated by the grammars:

$$\mathcal{E}_{\mathrm{r}} \ni e_r ::= v \mid e \mid \langle e_1 | e_2 \rangle \qquad \mathcal{C}_{\mathrm{r}} \ni c_r ::= x \leftarrow e_r \mid x \xleftarrow{\$} lap_{e_r}(e_r) \mid c \mid \langle c_1 | c_2 \rangle$$

where $v \in \mathcal{V}_{\mathrm{r}}, e, e_1, e_2 \in \mathcal{E}, c, c_1, c_2 \in \mathcal{C}$. Values can now be also pairs of unary values, that is $\mathcal{V}_{\mathrm{r}} \equiv \mathcal{V} \cup \mathcal{V}^2$.

To define the semantics for RPFOR, we first extend memories to allow program variables to map to pairs of integers, and array variables to map to pairs of arrays. In the following we will use the following projection functions $\lfloor \cdot \rceil_i$ for $i \in \{1, 2\}$, which project, respectively, the first (left) and second (right) elements of a pair construct (i.e., $\lfloor \langle c_1 | c_2 \rangle \rceil_i = c_i$, $\lfloor \langle e_1 | e_2 \rangle \rceil_i = e_i$ with $\lfloor v \rceil_i = v$ when $v \in \mathcal{V}$), and are homomorphic for other constructs.

The semantics of expressions in RPFOR is specified through the following judgment $\langle m_1, m_2, e, p_1, p_2 \rangle \downarrow_{\mathrm{rc}} \langle v, p_1', p_2' \rangle$, where $m_1, m_2 \in \mathcal{M}, p_1, p_2, p_1', p_2' \in P, e \in \mathcal{E}_{\mathrm{r}}, v \in \mathcal{V}_{\mathrm{r}}$. Similarly, for commands, we have the following judgment $\langle m_1, m_2, c, p_1, p_2 \rangle \rightarrow_{\mathrm{rc}} \langle m_1', m_2', c', p_1', p_2' \rangle$. Again, we use the predicate $\mathbf{Final}(\cdot)$ for configurations $\langle m_1, m_2, c, p_1, p_2 \rangle$ such that $c = \mathtt{skip}$, and lift the predicate to sets of configurations as well. Intuitively a relational probabilistic concrete configuration $\langle m_1, m_2, c, p_1, p_2 \rangle$ denotes a pair of probabilistic concrete states, that is a pair of subdistributions over the space of concrete memories. In Figure 5 a selection of the rules defining the judgments is presented. Most of the rules are quite natural. Notice how branching instructions combine both probabilistic and relational nondeterminism.

**r-if-conc-conc-true-false**

$$\frac{\langle m_1, m_2, e, p_1, p_2 \rangle \downarrow_{\mathrm{rc}} \langle v, p_1', p_2' \rangle \qquad \lfloor v \rceil_1, \lfloor v \rceil_2 \in \mathbb{Z} \qquad \lfloor v \rceil_1 > 0 \qquad \lfloor v \rceil_2 \leq 0}{\langle m_1, m_2, \mathtt{if}\ e\ \mathtt{then}\ c_1\ \mathtt{else}\ c_2, p_1, p_2 \rangle \rightarrow_{\mathrm{rc}} \langle m_1, m_2, \langle \lfloor c_1 \rceil_1 | \lfloor c_2 \rceil_2 \rangle, p_1', p_2' \rangle}$$

**r-if-prob-prob-true-false**

$$\frac{\langle m_1, m_2, e, p_1, p_2 \rangle \downarrow_{\mathrm{rc}} \langle v, p_1', p_2' \rangle \qquad \lfloor v \rceil_1, \lfloor v \rceil_2 \in \mathcal{X}_p}{\begin{array}{c}\langle m_1, m_2, \mathtt{if}\ e\ \mathtt{then}\ c_1\ \mathtt{else}\ c_2, p_1, p_2 \rangle \rightarrow_{\mathrm{rc}} \\ \langle m_1, m_2, \langle \lfloor c_1 \rceil_1 | \lfloor c_2 \rceil_2 \rangle, \lfloor v \rceil_1 > 0 @ p_1', \lfloor v \rceil_2 \leq 0 @ p_2' \rangle\end{array}}$$

**r-pair-step**

$$\frac{\begin{array}{c}\{i, j\} = \{1, 2\} \qquad \langle \lfloor m \rceil_i, c_i, p_i \rangle \rightarrow_{\mathrm{c}} \langle m_i', c_i', p_i' \rangle \\ c_j' = c_j \qquad p_j' = p_j \qquad m_j' = \lfloor m \rceil_j\end{array}}{\langle m_1, m_2, \langle c_1 | c_2 \rangle, p_1, p_2 \rangle \rightarrow_{\mathrm{rc}} \langle m_1', m_2', \langle c_1' | c_2' \rangle, p_1', p_2' \rangle}$$

Fig. 5: RPFOR selected rules

So, as in the case of PFOR, we collect sets of relational configurations using the judgment $\mathscr{R} \Rightarrow_{\mathrm{rc}} \mathscr{R}'$ with $\mathscr{R}, \mathscr{R}' \in \mathcal{P}(\mathcal{M} \times \mathcal{M} \times \mathcal{C}_{\mathrm{r}} \times P \times P)$, defined by only one rule:

**SUB-PDISTR-STEP**

$$\frac{\begin{array}{c}\langle m_1, m_2, c, p_1, p_2\rangle \in \mathscr{R} \\ \mathscr{R}_t \equiv \{\langle m_1', m_2', c', p_1', p_2'\rangle \mid \langle m_1, m_2, c, p_1, p_2\rangle \rightarrow_{\mathrm{rc}} \langle m_1', m_2', c', p_1', p_2'\rangle\} \\ \mathscr{R}' \equiv \Big(\mathscr{R} \setminus \{\langle m_1, m_2, c, p_1, p_2\rangle\}\Big) \cup \mathscr{R}_t\end{array}}{\mathscr{R} \Rightarrow_{\mathrm{rc}} \mathscr{R}'}$$

This rule picks and remove non deterministically one relational configuration from a set and adds to it all those configurations that are reachable from it. As mentioned before a run of a program in RPFOR corresponds to the execution of two runs the program in PFOR. Before making this precise we extend projection functions to relational configurations in the following way: $\lfloor\langle m_1, m_2, c, p_1, p_2\rangle\rfloor_i = \langle m_i, c, p_i\rangle$, for $i \in \{1, 2\}$. Projection functions extend in the obvious way also to sets of relational configurations. We are now ready to state the following lemma relating the execution in RPFOR to the one in PFOR:

**Lemma 5.** *Let* $i \in \{1, 2\}$ *then* $\mathscr{R} \Rightarrow^*_{\mathrm{rc}} \mathscr{R}'$ *iff* $\lfloor\mathscr{R}\rfloor_i \Rightarrow^*_{\mathrm{c}} \lfloor\mathscr{R}'\rfloor_i$.

### 5 Symbolic languages

In this section we lift the concrete languages, presented in the previous section, to their symbolic versions (respectively, SPFOR and SRPFOR) by extending them with symbolic values $X \in \mathcal{X}$. We use intentionally the same metavariables for symbolic values in $\mathcal{X}$ and $\mathcal{X}_p$ since they both represent symbolic values of some sort. However, we assume $\mathcal{X}_p \cap \mathcal{X} = \emptyset$ - this is because we want symbolic values in $\mathcal{X}$ to denote only unknown sets of integers, rather than sets of probability distributions. So, the meaning of $X$ should then be clear from the context.

#### 5.1 SPFOR

SPFOR expressions extend PFOR expressions with symbolic values $X \in \mathcal{X}$ Commands in SPFOR are the same as in PFOR but now symbolic values can appear in expressions.

In order to collect constraints on symbolic values we extend configurations with set of constraints over integer values, drawn from the set $\mathcal{S}$ (Figure 6a), not to be confused with probabilistic path constraints (Figure 6b). The former express constraints over integer values, for instance parameters of the distributions. In particular constraint expressions include standard arithmetic expressions with values being symbolic or integer constants, and array selection. Probabilistic path constraints now can also contain symbolic integer values. Hence,

$$\mathcal{S}_e \ni e ::= n \mid X \mid i \mid e \oplus e \mid |e|$$
$$\mathbf{store}(e,e,e) \mid \mathbf{select}(e,e)$$
$$\mathcal{S} \ni s ::= \top \mid e \circ e \mid s \wedge s \mid \neg s \mid \forall i.s$$

(a) Symbolic constraints. $X \in \mathcal{X}, n \in \mathbf{V}$.

$$sra ::= Y \xleftarrow{\$} lap_{c_e}(c_e)$$
$$sre ::= n \mid X \mid Y \mid re \oplus re$$
$$SP ::= Y = re \mid re > 0 \mid re \leq 0 \mid ra$$

(b) Prob. constraints. $c_e \in \mathcal{S}, X \in \mathcal{X}, Y \in \mathcal{X}_p$

Fig. 6: Grammar of constraints probabilistic path constraints now can be symbolic. This is needed to address examples branching on probabilistic values, such as the Above Threshold algorithm we discussed in Section 2.

Memories can now contain symbolic values and we represent arrays in memory as pairs $(X, v)$, where $v$ is a (concrete or symbolic) integer value representing the length of the array, and $X$ is a symbolic value representing the array content. The content of the arrays is kept and refined in the set of constraints by means of the $\mathbf{select}(\cdot,\cdot)$ and $\mathbf{store}(\cdot,\cdot,\cdot)$ operations. The semantics of expressions is captured by the judgment $(m, e, p, s) \downarrow_{\text{SP}} (v, p', s')$ including now a set of constraints over integers. The rules of the judgment are fully described in the extended version [11]. We briefly describe a selection of the rules. Rule **S-P-Op-2** applies when an arithmetic operation has both of its operands that reduce respectively to elements in $\mathcal{X}_p$. Appropriately it updates the set of probabilistic constraints. Rules **S-P-Op-5** instead fires when one of them is an integer and the other is a symbolic value. In this case only the list of symbolic constraints needs to be updated. Finally, in rule **S-P-Op-6** one of the operands reduces to an element in $\mathcal{X}_p$ and the other to an element in $\mathcal{X}$. We only update the list of probabilistic constraints appropriately, as integer constraints cannot contain symbols in $\mathcal{X}_p$.

The semantics of commands of SPFOR is described by small step semantics judgments of the form: $(m, c, p, s) \rightarrow_{\text{SP}} (m', c', p', s')$, including a set of constraints over integers. We provide a selection of the rules in Figure 7. Rule **S-P-If-sym-true** fires when a branching instruction is to be executed and the guard is reduced to either an integer or a value in $\mathcal{X}$, denoted by the set $\mathbf{V}_{\text{is}}$. In this case we can proceed with the true branch recording in the set of integer constraints the fact that the guard is greater than 0. Rule **S-P-If-prob-false** handles a branching instruction which has a guard reducing to a value in $\mathcal{X}_p$. In this case we can proceed in both branches, even though here we only show one of the two rules, by recording the conditioning fact on the list of probabilistic constraints. Finally, rule **S-P-Lap-Ass** handles probabilistic assignment. After having reduced both the expression for the mean and the expression for the scale to values we check that those are both either integers or symbolic integers, if that's the case we make sure that the scale is greater than 0 and we add a probabilistic constraints recording the fact that the modified variable now points to a probabilistic symbolic value related to a Laplace distribution.

The semantics of SPFOR has two sources of nondeterminism, from guards which reduce to symbolic values, and from guards which reduce to a probabilistic symbolic value. The collecting semantics of SPFOR, specified by judgments as

**S-P-If-sym-true**
$$\frac{(m, e, p, s) \downarrow_{\mathrm{SP}} (v, p', s') \qquad v \in \mathbf{V}_{\mathrm{is}}}{(m, \mathtt{if}\ e\ \mathtt{then}\ c_{tt}\ \mathtt{else}\ c_{ff}, p, s) \rightarrow_{\mathrm{SP}} (m, c_{tt}, p', s' \cup \{v > 0\})}$$

**S-P-If-prob-false**
$$\frac{(m, e, p, s) \downarrow_{\mathrm{SP}} (v, p', s') \qquad v \in \mathcal{X}_p}{(m, \mathtt{if}\ e\ \mathtt{then}\ c_{tt}\ \mathtt{else}\ c_{ff}, p, s) \rightarrow_{\mathrm{SP}} (m, c_{ff}, p'@[v \leq 0], s')}$$

**S-P-Lap-Ass**
$$\frac{\begin{array}{c}(m, e_a, p, s) \downarrow_{\mathrm{SP}} (v_a, p', s') \qquad (m, e_b, p', s') \downarrow_{\mathrm{SP}} (v_b, p'', s'') \qquad X\ \mathbf{fresh}(\mathcal{X}_p) \\ v_a, v_b \in \mathbf{V}_{\mathrm{is}} \qquad s''' = s'' \cup \{v_b > 0\} \qquad p''' = p''@[X \xleftarrow{\$} lap_{v_b}(v_a)]\end{array}}{(m, x \xleftarrow{\$} lap_{e_b}(e_a), p, s) \rightarrow_{\mathrm{SP}} (m[x \mapsto X], \mathtt{skip}, p''', s''')}$$

Fig. 7: SPFOR: Semantics of commands (selected rules)

$\mathcal{H} \Rightarrow_{\mathrm{sp}} \mathcal{H}'$ (for sets of configurations $\mathcal{H}$ and $\mathcal{H}'$) takes care of both of them. The rule for this judgment form is:

**s-p-collect**
$$\frac{\begin{array}{c}\mathcal{D}_{[s]} \subseteq \mathcal{H} \qquad \mathcal{H}' \equiv \{(m', c', p', s') \mid \exists (m, c, p, s) \in \mathcal{D}_{[s]} \text{ s.t.} \\ (m, c, p, s) \rightarrow_{\mathrm{SP}} (m', c', p', s') \wedge \mathbf{SAT}(s')\}\end{array}}{\mathcal{H} \Rightarrow_{\mathrm{sp}} \big(\mathcal{H} \setminus \mathcal{D}_{[s]}\big) \cup \mathcal{H}'}$$

Unlike in the deterministic case of the rule **Set-Step**, where only one configuration was chosen nondeterministically from the initial set, here we select nondeterministically a (maximal) set of configurations all sharing the same symbolic constraints. The notation $\mathcal{D}_{[s]} \subseteq \mathcal{H}$ means that $\mathcal{D}$ is the maximal subset of configuration in $\mathcal{H}$ which have $s$ as set of constraints. We use $\mathcal{H} \overset{\mathcal{D}_{[s]}}{\underset{\mathrm{sp}}{\Longrightarrow}} \mathcal{H}'$ when we want to make explicit the set of symbolic configurations, $\mathcal{D}_{[s]}$, that we are using to make the step. Intuitively, **s-p-collect** starts from a set of configurations and reaches all of those that are reachable from it - all the configurations that have a satisfiable set of constraints and are reachable from one of the original configurations with only one step of the symbolic semantics. Notice that in a set of constraints we can have constraints involving probabilistic symbols, e.g. if the i-th element of an array is associated with a random expression. Nevertheless, the predicate $\mathbf{SAT}(\cdot)$ does not need to take into consideration relations involving probabilistic symbolic constraints but only relations involving symbolic values denoting integers. The following lemma of coverage connects PFOR with SPFOR ensuring that a concrete execution is covered by a symbolic one.

**Lemma 6 (Probabilistic Unary Coverage).** *If $\mathcal{H} \overset{\mathcal{D}_{[s]}}{\underset{\mathrm{sp}}{\Longrightarrow}} \mathcal{H}'$ and $\sigma \models_{\mathcal{I}} \mathcal{D}_{[s]}$ then $\exists \sigma', \mathcal{D}_{[s']} \subseteq \mathcal{H}'$ such that $\sigma' \models_{\mathcal{I}} \mathcal{D}_{[s']}$, and $\sigma(\mathcal{D}_{[s]}) \Rightarrow_{\mathrm{p}}^{*} \sigma'(\mathcal{D}_{[s']})$.*

### 5.2 SRPFOR

The language presented in this section is the the symbolic extension of the concrete language RPFOR. It can also be seen as the relational extension of SPFOR.

The key part of this language's semantics will be the handling of the probabilistic assignment. For that construct we will provide 2 rules instead of one. The first one is the obvious one which carries on a standard symbolic probabilistic assignment. The second one will implement a coupling semantics. The syntax of the SRPFOR, presented in Figure 8, extends the syntax of RPFOR by adding symbolic values. The main change is in the grammar of expressions, while the syntax for commands is almost identical to that of RPFOR.

$$\mathcal{E}_{\mathrm{rs}} \ni e_{sr} ::= e_s \mid \langle e_s | e_s \rangle \mid e_{sr} \oplus e_{sr} \mid a[e_{sr}]$$
$$\mathcal{C}_{\mathrm{rs}} \ni c_{sr} ::= c_s \mid \langle c_s | c_s \rangle \mid c_{sr};c_{sr} \mid x \leftarrow e_{sr} \mid a[e_{sr}] \leftarrow e_{sr} \mid x \xleftarrow{\$} lap_{e_s}(e_{sr}) \mid \texttt{if } e_{sr} \texttt{ then } c_{sr} \texttt{ else } c_{sr} \mid \texttt{for } (x \texttt{ in } e_{sr}{:}e_{sr}) \texttt{ do } c_{sr} \texttt{ od}$$

Fig. 8: SRPFOR syntax. $e_s \in \mathcal{E}_{\mathrm{s}}, c_s \in \mathcal{C}_{\mathrm{s}}$.

As in the case of RPFOR, only unary symbolic expressions and commands are admitted in the pairing construct. This invariant is maintained by the semantics rules. As for the other languages, we provide a big-step evaluation semantics for expressions whose judgments are of the form $(m_1, m_2, e, p_1, p_2, s) \downarrow_{\mathrm{SRP}} (v, p_1', p_2', s')$. The only rule defining the judgment $\downarrow_{\mathrm{SRP}}$ is **S-R-P-Lift** and it is presented in the extended version [11]. The rule projects the symbolic relational expression first on the left and evaluates it to a unary symbolic value, potentially updating the probabilistic symbolic constraints and the symbolic constraints. It then does the same projecting the expression on the right but starting from the potentially previously updated constraints. Now, the only case when the value returned is unary is when both the previous evaluation returned equal integers, in all the other cases a pair of values is returned. So, the relational symbolic semantics leverages the unary semantics. For the semantics of commands we use the following evaluation contexts to simplify the exposition:

$$\mathcal{CTX} ::= [\,\cdot\,] \mid \mathcal{CTX};c$$
$$\mathcal{P} ::= \langle \cdot;c | \cdot \rangle \mid \langle \cdot | \cdot ;c \rangle \mid \langle \cdot | \cdot \rangle \mid \langle \cdot;c | \cdot ;c \rangle$$

Notice how $\mathcal{P}$ gets saturated by pairs of commands. Moreover, we separate commands in two classes. We call *synchronizing* all the commands in $\mathcal{C}_{\mathrm{rs}}$ with the following shapes $x \xleftarrow{\$} lap_{e_2}(e_1)$, $\langle x \xleftarrow{\$} lap_{e_2}(e_1) | x' \xleftarrow{\$} lap_{e_2'}(e_1') \rangle$, since they allow synchronization of two runs using coupling rules. We call non synchronizing all the other commands.

**Semantics of non synchronizing commands** We consider judgments of the form $(m_1, m_2, c, p_1, p_2, s) \to_{\mathrm{SRP}} (m_1', m_2', c', p_1', p_2', s')$ and a selection of the rules is given in Figure 9. An explanation of the rules follows. Rule **s-r-if-prob-prob-true-false** fires when evaluating a branching instruction. In particular, it fires when the guard evaluates on both side to a probabilistic symbolic value. In this case the semantics can continue with the true branch on the left run and

**s-r-if-prob-prob-true-false**

$$\frac{(m_1, m_2, e, p_1, p_2, s) \downarrow_{\mathrm{SRP}} (v, p_1', p_2', s') \qquad \lfloor v \rfloor_1, \lfloor v \rfloor_2 \in \mathcal{X}_p \qquad p_1'' \equiv p_1'@[\lfloor v \rfloor_1 > 0] \qquad p_2'' \equiv p_2'@[\lfloor v \rfloor_2 \leq 0]}{(m_1, m_2, \texttt{if } e \texttt{ then } c_{tt} \texttt{ else } c_{\mathit{ff}}, p_1, p_2, s) \to_{\mathrm{SRP}} (m_1, m_2, \langle \lfloor c_{tt} \rfloor_1 | \lfloor c_{\mathit{ff}} \rfloor_2 \rangle, p_1'', p_2'', s')}$$

**s-r-if-prob-sym-true-false**

$$\frac{(m_1, m_2, e, p_1, p_2, s) \downarrow_{\mathrm{SRP}} (v, p_1', p_2', s') \qquad \lfloor v \rfloor_1 \in \mathcal{X}_p \; \lfloor v \rfloor_2 \in \mathcal{X} \qquad p_1'' \equiv p_1'@[\lfloor v \rfloor_1 > 0] \qquad s''' \equiv s'' \cup \{\lfloor v \rfloor_2 \leq 0\}}{(m_1, m_2, \texttt{if } e \texttt{ then } c_{tt} \texttt{ else } c_{\mathit{ff}}, p_1, p_2, s) \to_{\mathrm{SRP}} (m_1, m_2, c_{tt}, p_1'', p_2', s''')}$$

**s-r-pair-lap-skip**

$$\frac{(m_1, x \xleftarrow{\$} lap_{e_b}(e_a), p_1, s) \to_{\mathrm{SP}} (m_1', \texttt{skip}, p_1', s')}{(m_1, m_2, \langle x \xleftarrow{\$} lap_{e_b}(e_a) | \texttt{skip} \rangle, p_1, p_2, s) \to_{\mathrm{SRP}} (m_1', m_2, \langle \texttt{skip} | \texttt{skip} \rangle, p_1', p_2, s')}$$

**s-r-pair-lapleft-sync**

$$\frac{c \not\equiv x \xleftarrow{\$} lap_{e_b'}(e_a') \qquad \mathcal{P} \equiv \langle \cdot | \cdot \rangle \qquad (m_2, c, p_2, s) \to_{\mathrm{SP}} (m_2', c', p_2', s')}{(m_1, m_2, \mathcal{P}(x \xleftarrow{\$} lap_{e_b}(e_a), c), p_1, p_2, s) \to_{\mathrm{SRP}} (m_1, m_2', \langle x \xleftarrow{\$} lap_{e_b}(e_a) | c' \rangle, p_1, p_2', s')}$$

**s-r-pair-ctxt-1**

$$\frac{x \xleftarrow{\$} lap_{e_b}(e_a) \notin \{c_1, c_2\} \qquad |\{c_1, c_2\}| = 2 \qquad \{1, 2\} = \{i, j\} \qquad c_i' \equiv c_i \qquad p_i' \equiv p_i \qquad m_i' \equiv m_i \qquad (m_j, c_j, p_j, s) \to_{\mathrm{SP}} (m_j', c_j', p_j', s')}{(m_1, m_2, \mathcal{P}(c_1, c_2), p_1, p_2, s) \to_{\mathrm{SRP}} (m_1', m_2', \mathcal{P}(c_1', c_2'), p_1', p_2', s')}$$

**s-r-pair-ctxt-2**

$$\frac{\mathcal{P} \not\equiv \langle \cdot | \cdot \rangle \qquad (m_1, m_2, \langle c_1 | c_2 \rangle, p_1, p_2, s) \to_{\mathrm{SRP}} (m_1', m_2', \langle c_1' | c_2' \rangle, p_1', p_2', s')}{(m_1, m_2, \mathcal{P}(c_1, c_2), p_1, p_2, s) \to_{\mathrm{SRP}} (m_1', m_2', \mathcal{P}(c_1', c_2'), p_1', p_2', s')}$$

Fig. 9: SRPFOR: Semantics of non synchronizing commands. Selected rules.

with the false branch on the right one. Notice that commands are projected to avoid pairing commands appearing in a nested form. Rule **s-r-if-prob-sym-true-false** applies when the guard of a branching instruction evaluates to a probabilistic symbolic value on the left run and a symbolic integer value on the right one. The rule allows to continue on the true branch on the left run and on the false branch on the right one. Notice that in one case the probabilistic list of constraints is updated, while on the other the symbolic set of constraints.

Rule **s-r-pair-lap-skip** handles the pairing command where on the left hand side we have a probabilistic assignment and on the right a skip instruction. In this case, there is no *hope for synchronization* between the two runs and hence we can just perform the left probabilistic assignment relying on the unary symbolic semantics. Rule **s-r-pair-lapleft-sync** instead applies when on the left we have a probabilistic assignment and on the right we have another arbitrary command. In this case we can hope to reach a situation where on the right run another probabilistic assignment appears. Hence, it makes sense to continue the computation in a unary way on the right side. Again $\rightarrow_{\text{SRP}}$ is a nondeterministic semantics. The nondeterminism comes from the use of probabilistic symbols and symbolic values as guards, and by the relational approach. So, in order to collect all the possible traces stemming from such nondeterminism we define a collecting semantics relating set of configurations to set of configurations.

The semantics is specified through a judgment of the form: $\mathscr{SR} \Rightarrow_{\text{srp}} \mathscr{SR}'$, with $\mathscr{SR}, \mathscr{SR}' \in \mathcal{P}(\boldsymbol{\mathcal{M}}_{\text{SP}} \times \boldsymbol{\mathcal{M}}_{\text{SP}} \times \mathcal{C}_{\text{rs}} \times SP \times SP \times \mathcal{S})$. The only rule defining the judgment is the following natural lifting of the one for the unary semantics.

**s-r-p-collect**

$$\frac{\begin{array}{c}\mathscr{R}_{[s]} \subseteq \mathscr{SR} \qquad \mathscr{SR}' \equiv \{(m_1', m_2', c', p_1', p_2', s') \mid \\ \exists (m_1, m_2, c, p_1, p_2, s) \in \mathscr{R}_{[s]} \text{ s.t. } (m_1, m_2, c, p_1, p_2, s) \rightarrow_{\text{SRP}} (m_1', m_2', c', p_1', p_2', s') \\ \wedge \mathbf{SAT}(s')\}\end{array}}{\mathscr{SR} \Rightarrow_{\text{srp}} \left(\mathscr{SR} \setminus \mathscr{R}_{[s]}\right) \cup \mathscr{SR}'}$$

The rule, and the auxiliary notation $\mathscr{R}_{[s]}$, is pretty similar to that of $\mathsf{SPFOR}$, the only difference is that here sets of symbolic relational probabilistic configurations are considered instead of symbolic (unary) probabilistic configurations.

**Semantics of synchronizing commands** We define a new judgment with form $\mathscr{G} \rightsquigarrow \mathscr{G}'$, with $\mathscr{G}, \mathscr{G}' \in \mathcal{P}(\mathcal{P}(\boldsymbol{\mathcal{M}}_{\text{SP}} \times \boldsymbol{\mathcal{M}}_{\text{SP}} \times \mathcal{C}_{\text{rs}} \times SP \times SP \times \mathcal{S}))$. In Figure 10, we give a selection of the rules. Rule **Proof-Step-No-Sync** applies when no synchronizing commands are involved, and hence there is no possible coupling rule to be applied. In the other rules, we use the variable $\epsilon_c$ to symbolically count the privacy budget in the current relational execution. The variable gets increased when the rule **Proof-Step-Lap-Gen** fires. This symbolic counter variable is useful when trying to prove equality of certain variables without spending more than a specific budget. This rule is the one we can use in most cases when we need to reason about couplings on the Laplace distributions. In the set of sets of configurations $\mathscr{G}$, a set of configurations, $\mathscr{SR}$, is nondeterministically chosen. Among elements in $\mathscr{SR}$ a configuration is also nondeterministically chosen. Using contexts we check that in the selected configuration the next command to execute is the probabilistic assignment. After reducing to values both the mean and scale expression, and verified (that is, assumed in the set of constraints) that in the two runs the scales have the same value, the rule adds to the set of constraints a new element, that is, $E'' = E' + |\lfloor v_a \rfloor_1 - \lfloor v_a \rfloor_2| \cdot K'$, where $K, K', E''$ are fresh symbols denoting integers and $E'$ is the symbolic integer to which the budget variable $\epsilon_c$ maps to. Notice that $\epsilon_c$ needs to point to the same symbol in both memories. This is because it is a shared variable tracking the privacy budget spent so far in both runs. This new constraint increases the budget spent. The other constraint added is the real coupling relation, that is $X_1 + K = X_2$. Where $X_1, X_2$ are fresh in $\mathcal{X}$. Later, $K$ will be existentially quantified in order to search for a proof of $\epsilon$-indistinguishability.

Rule **Proof-Step-Avoc** does not use any coupling rule but treats the samples in a purely symbolic manner. It intuitively asserts that the two samples are

**Proof-Step-No-Sync**

$$\frac{\mathscr{SR} \in \mathscr{G} \qquad \mathscr{SR} \Rightarrow_{\text{srp}} \mathscr{SR}' \qquad \mathscr{G}' \equiv \big(\mathscr{G} \setminus \{\mathscr{SR}\}\big) \cup \{\mathscr{SR}'\}}{\mathscr{G} \rightsquigarrow \mathscr{G}'}$$

**Proof-Step-No-Coup**

$$\frac{\begin{array}{c}(m_1, m_2, \mathcal{CTX}[x \xleftarrow{\$} lap_{e_b}(e_a)], p_1, p_2, s) \in \mathscr{SR} \in \mathscr{G} \\ (m_1, m_2, e_a, p_1, p_2, s) \quad \downarrow_{\text{SRP}} (v_a, p_1', p_2', s_a) \\ (m_1, m_2, e_b, p_1', p_2', s_a) \quad \downarrow_{\text{SRP}} (v_b, p_1'', p_2'', s_b) \\ X_1, X_2\, \mathbf{fresh}(\mathcal{X}_p) \qquad m_1' \equiv m_1[x \mapsto X_1] \qquad m_2' \equiv m_2[x \mapsto X_2] \\ p_1''' \equiv p_1''@[X_1 \xleftarrow{\$} lap_{\lfloor v_b \rfloor_1}(\lfloor v_a \rfloor_1)] \qquad p_2''' \equiv p_2''@[X_2 \xleftarrow{\$} lap_{\lfloor v_b \rfloor_2}(\lfloor v_a \rfloor_2)] \\ \mathscr{SR}' \equiv \Big(\mathscr{SR} \setminus \{(m_1, m_2, \mathcal{CTX}[x \xleftarrow{\$} lap_{e_b}(e_a)], p_1, p_2, s)\}\Big) \cup \\ \{(m_1', m_2', \mathcal{CTX}[\texttt{skip}], p_1''', p_2''', s'')\} \qquad \mathscr{G}' \equiv \Big(\mathscr{G} \setminus \{\mathscr{SR}\}\Big) \cup \{\mathscr{SR}'\}\end{array}}{\mathscr{G} \rightsquigarrow \mathscr{G}'}$$

**Proof-Step-Avoc**

$$\frac{\begin{array}{c}(m_1, m_2, \mathcal{CTX}[x \xleftarrow{\$} lap_{e_b}(e_a)], p_1, p_2, s) \in \mathscr{SR} \in \mathscr{G} \\ (m_1, m_2, e_a, p_1, p_2, s) \downarrow_{\text{SRP}} (v_a, p_1', p_2', s_a) \\ (m_1, m_2, e_b, p_1', p_2', s_a) \downarrow_{\text{SRP}} (v_b, p_1'', p_2'', s_b) \\ X_1, X_2\, \mathbf{fresh}(\mathcal{X}) \qquad m_1' \equiv m_1[x \mapsto X_1] \qquad m_2' \equiv m_2[x \mapsto X_2] \\ \mathscr{G}' \equiv \big(\mathscr{G} \setminus \{\mathscr{SR}\}\big) \cup \{\mathscr{SR}'\} \\ \mathscr{SR}' \equiv \big(\mathscr{SR} \setminus \{(m_1, m_2, \mathcal{CTX}[x \xleftarrow{\$} lap_{e_b}(e_a)], p_1, p_2, s)\}\big) \\ \cup \{(m_1', m_2', \mathcal{CTX}[\texttt{skip}], p_1'', p_2'', s'')\}\end{array}}{\mathscr{G} \rightsquigarrow \mathscr{G}'}$$

**Proof-Step-Lap-Gen**

$$\frac{\begin{array}{c}(m_1, m_2, \mathcal{CTX}[x \xleftarrow{\$} lap_{e_b}(e_a)], p_1, p_2, s) \in \mathscr{SR} \in \mathscr{G} \\ (m_1, m_2, e_a, p_1, p_2, s) \downarrow_{\text{SRP}} (v_a, p_1', p_2', s_a) \\ (m_1, m_2, e_b, p_1', p_2', s_a) \downarrow_{\text{SRP}} (v_b, p_1'', p_2'', s_b) \\ s' \equiv s_b \cup \{\lfloor v_b \rfloor_1 = \lfloor v_b \rfloor_2, \lfloor v_b \rfloor_1 > 0\} \qquad m_1(\epsilon_c) = E' = m_2'(\epsilon_c) \\ E'', X_1, X_2, K, K'\, \mathbf{fresh}(\mathcal{X}) \qquad m_1' \equiv m_1[x \mapsto X_1][\epsilon_c \mapsto E''] \\ m_2' = m_2[x \mapsto X_2][\epsilon_c \mapsto E''] \qquad m(\epsilon) = E \\ s'' \equiv s' \cup \{X_1 + K = X_2, K \leq K', K' \cdot E = \lfloor v_b \rfloor_1, \\ E'' = E' + |\lfloor v_a \rfloor_1 - \lfloor v_a \rfloor_2| \cdot K'\} \\ p_1''' \equiv p_1''@[X_1 \xleftarrow{\$} lap_{\lfloor v_b \rfloor_1}(\lfloor v_a \rfloor_1)] \qquad p_2''' \equiv p_2''@[X_2 \xleftarrow{\$} lap_{\lfloor v_b \rfloor_2}(\lfloor v_a \rfloor_2)] \\ \mathscr{G}' \equiv \big(\mathscr{G} \setminus \{\mathscr{SR}\}\big) \cup \{\mathscr{SR}'\} \\ \mathscr{SR}' \equiv \big(\mathscr{SR} \setminus \{(m_1, m_2, \mathcal{CTX}[x \xleftarrow{\$} lap_{e_b}(e_a)], p_1, p_2, s)\}\big) \\ \cup \{(m_1', m_2', \mathcal{CTX}[\texttt{skip}], p_1''', p_2''', s'')\}\end{array}}{\mathscr{G} \rightsquigarrow \mathscr{G}'}$$

Fig. 10: SRPFOR: Proof collecting semantics, selected rules drawn from the distributions and assigns to them arbitrary integers free to vary on the all domain of the Laplace distribution.

Finally, rule **Proof-Step-No-Coup** applies to synchronizing commands as well. It does not add any relational constraints to the samples. This rules intuitively means that we are not correlating in any way the two samples. Notice that since we are not using any coupling rule we don't need to check that the scale value is the same in the two runs as it is requested in the previous rule. We could think of this as a way to encode the relational semantics of the program in an expression which later can be fed in input to other tools.

The main difference with the previous rule is that here we treat the sampling instruction symbolically and that is why the fresh symbols are in $\mathcal{X}_p$, denoting subdistributions, rather than in $\mathcal{X}$, denoting sampled integers. When the program involves a synchronizing command we basically fork the execution when it is time to execute it. The set of configurations allow us to explore different paths, one for every rule applicable.

### 6 Metatheory

The coverage lemma can be extended also to the relational setting.

**Lemma 7 (Probabilistic Relational Coverage).** *If* $\mathcal{SR} \xRightarrow[\text{srp}]{\mathcal{R}_{[s]}} \mathcal{SR}'$ *and* $\sigma \models_{\mathcal{I}} \mathcal{R}_{[s]}$ *then* $\exists \sigma', \mathcal{R}_{[s']} \in \mathcal{SR}'$ *such that* $\mathcal{R}_{[s']} \subseteq \mathcal{SR}', \sigma' \models_{\mathcal{I}} \mathcal{R}_{[s']}$*, and* $\sigma(\mathcal{R}_{[s]}) \Rightarrow^{*}_{\text{rp}} \sigma'(\mathcal{R}_{[s']})$.

This can also be extended to $\rightsquigarrow$ if we consider only the fragment that only uses the rules **Proof-Step-No-Sync**, and **Proof-Step-No-Coupl**.

The language of relational assertions $\Phi, \Psi \dots$ is defined using first order predicate logic formulas involving relational program expressions and logical variables in $\mathsf{LogVar}$. The interpretation of a relational assertions is naturally defined as a subset of $\mathcal{M}_{\text{c}} \times \mathcal{M}_{\text{c}}$, the set of pairs of memories modeling the assertion. We will denote by $[\![\cdot]\!]_{\cdot}$ the substitution function mapping the variables in an assertion to the values they have in a memory (unary or relational). More details are in [10].

**Definition 4.** *Let* $\Phi, \Psi$ *be relational assertions,* $c \in \mathcal{C}_{\text{r}}$*,* $\mathcal{I} : \mathsf{LogVar} \to \mathbb{R}$ *be an interpretation defined on* $\epsilon$*. We say that,* $\Phi$ *yields* $\Psi$ *through* $c$ *within* $\epsilon$ *under* $\mathcal{I}$ *(and we write* $\mathcal{I} \vdash c : \Phi \xrightarrow{\epsilon} \Psi$*) iff*

1. $\{\{\{\langle m_{I_1}, m_{I_2}, c, [], [], [\![\Phi]\!]_{m_I} \rangle\}\}\} \rightsquigarrow^{*} \mathcal{G}$
2. $\exists \mathcal{H}_{\text{sr}} = \{\mathcal{H}s_1, \dots, \mathcal{H}s_t\} \in \mathcal{G}$ *such that* ***Final***$(\mathcal{H}_{\text{sr}})$ *and* $\forall \langle m_1, m_2, \mathtt{skip}, p_1, p_2, s \rangle \in \bigcup_{\mathcal{D} \in \mathcal{H}_{\text{sr}}} \mathcal{D}.\ \exists \vec{k}.\ s \implies [\![\Psi \wedge \epsilon_c \leq \epsilon]\!]_{\langle m_1 | m_2 \rangle}$ *where* $m_I \equiv \langle m_{I_1} | m_{I_2} \rangle = \langle m'_{I_1}[\epsilon_c \mapsto 0] | m'_{I_2}[\epsilon_c \mapsto 0] \rangle$*,* $m'_{I_1}$*, and* $m'_{I_2}$ *are fully symbolic memories, and* $\vec{k} = k_1, k_2, \dots$ *are the symbols generated by the rules for synchronizing commands.*

The idea of this definition is to make the proof search automated. When proving differential privacy we will usually consider $\Psi$ as being equality of the output variables in the two runs and $\Phi$ as being our preconditions. We can now prove the soundness of our approach.

**Lemma 8 (Soundness).** *Let* $c \in \mathcal{C}_{\mathrm{r}}$. *If* $\mathcal{I} \vdash c : D_1 \sim D_2 \xrightarrow{\epsilon} o_1 = o_2$ *then c is* $\epsilon$*-differentially private.*

We can also prove the soundness of refutations obtained by the semantics.

**Lemma 9 (Soundness for refutation).** *Suppose that we have a reduction* $\{\{\{\langle m_1, m_2, c, [], [], [\![\Phi]\!]_{\langle m_1 | m_2 \rangle}\rangle\}\}\} \rightsquigarrow \mathscr{G}$, *and* $\mathscr{H}s \in \mathcal{H} \in \mathscr{G}$ *and,* $\exists \sigma \models_{\mathbb{Z}} s$ *such that* $\Delta_\epsilon([\![\lfloor c \rfloor_1]\!]_{\mathcal{C}}(\sigma(m_1)), [\![\lfloor c \rfloor_2]\!]_{\mathcal{C}}(\sigma(m_2))) > 0$ *then c is not differentially private.*

### 7 Strategies for counterexample finding

Lemma 9 is hard to use to find counterexamples in practice. For this reasons we will now describe three strategies that can help in reducing the effort in counterexample finding. These strategies help in isolating traces that could potentially lead to violations. For this we need first some notation. Given a set of constraints $s$ we define the triple $\Omega = \langle \Omega_1, \Omega_2, C(\vec{k})\rangle \equiv \langle \lfloor s \rfloor_1, \lfloor s \rfloor_2, s \setminus (\lfloor s \rfloor_1 \cup \lfloor s \rfloor_2)\rangle$. We sometimes abuse notation and consider $\Omega$ also as a set of constraints given by the union of its first, second and third projection, and we will also consider a set of constraints as a single proposition given by the conjunction of its elements. The set $C(\vec{k})$ contains relational constraints coming from either preconditions or invariants or, from the rule **Proof-Step-Lap-Gen**. The potentially empty vector $\vec{k} = K_1, \ldots K_n$ is the set of fresh symbols $K$ generated by that rule. In the rest of the paper we will assume the following simplifying assumption.

**Assumption 1** *Consider* $c \in \mathcal{C}_{\mathrm{r}}$ *with output variable o, then c is such that* $\{\{\{\langle m_1, m_2, c, [], [], s\rangle\}\}\} \rightsquigarrow^* \mathscr{G}$ *and* $\forall \mathscr{H}\langle \Omega_1, C(\vec{k}), \Omega_2\rangle \in \mathcal{H} \in \mathscr{G}.\textbf{\textit{Final}}(\mathcal{H}) \wedge o_1 = o_2 \implies \Omega_1 \Leftrightarrow \Omega_2$.

This assumption allow us to consider only programs for which it is necessary for the output variable on both runs to assume the same value, that the two runs follow the same branches. That is, if the two output differ then the two executions must have, at some point, taken different branches.

The following definition will be used to distinguish relational traces which are reachable on one run but not on the other. We call these traces *orthogonal*.

**Definition 5.** *A final relational symbolic trace is orthogonal when its set of constraints is such that* $\exists \sigma.\sigma \not\models \Omega_2$ *and* $\sigma \models \Omega_1 \wedge C(\vec{k})$. *That is a trace for which* $\neg(\Omega_1 \wedge C(\vec{k}) \implies \Omega_2)$ *is satisfiable.*

The next definition, instead, will be used to isolate relational traces for which it is not possible that the left one is executed but the right one is not. We call these traces *specular*.

**Definition 6.** *A final relational symbolic trace is specular if* $\exists \vec{k}.\Omega_1 \wedge C(\vec{k}) \implies \Omega_2$.

The constraint $\Omega_1 \land C(\vec{k})$ includes all the constraints coming from the left projection's branching of the symbolic execution and all the relational assumptions such as the adjacency condition, and all constraints added by the potentially fired **Proof-Step-Lap-Gen** rule. A specular trace is such that its left projection constraints plus the relational assumptions imply the right projection constraints. We will now describe our three strategies.

**Strategy A** In this strategy CRSE uses only the rule **Proof-Step-Avoc** for sampling instructions, also this strategy searches for orthogonal relational traces. Under assumption 1, if this happens for a program then it must be the case that the program can output one value on one run with some probability but the same value has 0 probability of being output on the second run. This implies that for some input the program has an unbounded privacy loss. To implement this strategy CRSE looks for orthogonal relational traces $\langle m_1, m_2, \texttt{skip}, p_1, p_2, \Omega\rangle$ such that: $\exists\sigma.\sigma \models \Omega_1 \land C(\vec{k})$ but $\sigma \not\models \Omega_2$. Notice that using this strategy $\vec{k}$ will always be empty, as the rule used for samplings does not introduce any coupling between the two samples.

**Strategy B** This strategy symbolically executes the program in order to find a specular trace for which no matter how we relate, within the budget, the various pairs of samples $X_1^i, X_2^i$ in the two runs - using the relational schema $X_1^i + K_i = X_2^i$ - the postcondition is always false. That is CRSE looks for specular relational traces $\langle m_1, m_2, \texttt{skip}, p_1, p_2, \Omega\rangle$ such that: $\forall\vec{k}.[(\Omega_1 \land C(\vec{k}) \implies \Omega_2) \land [\![\epsilon_c \le \epsilon)]\!]_{\langle m_1|m_2\rangle}] \implies [\![o_1 \neq o_2]\!]_{\langle m_1|m_2\rangle}$.

**Strategy C** This strategy looks for relational traces for which the output variable takes the same value on the two runs but too much of the budget was spent. That is CRSE looks for traces $\langle m_1, m_2, \texttt{skip}, p_1, p_2, \Omega\rangle$ such that: $\forall\vec{k}.[\Omega_1 \land C(\vec{k}) \land \Omega_2 \implies [\![o_1 = o_2]\!]_{\langle m_1|m_2\rangle}] \implies [\![\epsilon_c > \epsilon]\!]_{\langle m_1|m_2\rangle}$.

Of the presented strategies only strategy A is sound with respect to counterexample finding, while the other two apply when the algorithm cannot be proven differentially private by any combination of the rules. In this second case though, CRSE provides counterexamples which agree with other refutation oriented results in literature. This strategies are hence termed *useful* because they amount to heuristics that can be applied in some situations.

### 8 Examples

In this section we will review the examples presented in Section 2 and variations thereof to show how CRSE works.

**Unsafe Laplace mechanism: Algorithm 4.** This algorithm is not $\epsilon$-d.p because the noise is a constant and it is not calibrated to the sensitivity $r$ of the query $q$. This translates in any attempt based on coupling rules to use too much of the budget. This program has only one possible final relational trace:

$\langle m_1, m_2, \texttt{skip}, p_1, p_2, \langle \Omega_1, C(\vec{k}, \Omega_2)\rangle\rangle$. Since there are no branching instructions $\Omega_1 = \{\lfloor 2E \rfloor_1 > 0\}$ and $\Omega_2 = \emptyset$, where $m_1(\epsilon) = m_2(\epsilon) = E$. Since there is one sampling instruction $C(\vec{k})$ will include $\{|Q_{d1} - Q_{d2}| \leq R, \mathrm{P}_1 + K = \mathrm{P}_2, E_c =| K | \cdot K' \cdot E, O_1 = \mathrm{P}_1 + Q_{d1}, O_2 = \mathrm{P}_2 + Q_{d2}\}$, with $m_1(o) = O_1, m_2(o) = O_2, m_1(\epsilon_c) = m_2(\epsilon_c) = E_c, m_i(\rho_i) = \mathrm{P}_i$. Intuitively, given this set of constraints, if it has to be the case that $O_1 = O_2$ then, $Q_{d1} - Q_{d_2} = K$. But $Q_{d1} - Q_{d_2}$ can be $R$ and hence, $E_c$ is at least $R$. So, if we want to equate the two output we need to spend $r$ times the budget. Any relational input satisfying the precondition will give us a counterexample, provided the two projections are different.

**Algorithm 4**
A buggy Laplace mechanism

**Input:** $q\colon \mathcal{D} \to \mathbb{Z}$, $D : \mathcal{D}, \epsilon : \mathcal{R}^+$
**Output:** $o : \{true, false\}$
**Precondition**
$D_1 \sim D_2 \Rightarrow |q(D_1) - q(D_2)| \leq r$
**Postcondition** $o_1 = o_2$

```
1: v ← q(D)
2: ρ ←$ lap_ε(0)
3: o ← v + ρ
4: return o
```

**A safe Laplace mechanism.** By substituting line 2 in Algorithm 4 with $\rho \xleftarrow{\$} lap_{r*\epsilon}(0)$ we get an $\epsilon$-DP algorithm. Indeed when executing that line CRSE would generate the following constraint $\mathrm{P}_1 + K_0 = \mathrm{P}_2 \wedge \mid K_0 + 0 - 0 \mid \leq K_1 \wedge O_1 = V_1 + \mathrm{P}_1 \wedge O_2 = V_2 + \mathrm{P}_2$. Which by instantiating $K = 0, K_1 = V_2 - V_1$ implies $O_1 = O_2 \wedge E_c \leq E$.

**Unsafe sparse vector implementation: Algorithm 2.** We already discussed why this algorithm is not $\epsilon$-differentially private. Algorithm 2 satisfies Assumption 1 because it outputs the whole array $o$ which takes values of the form $\perp^i, t$ or $\perp^n$ for $1 \leq i \leq n$ and $t \in \mathbb{R}$. The array, hence, encodes the whole trace. So if two runs of the algorithm output the same value it must be the case that they followed the same branching instructions. Let's first notice that the algorithm is trivially $\epsilon$ differentially private, for any $\epsilon$, when the number of iterations $n$ is less than or equal to 4.

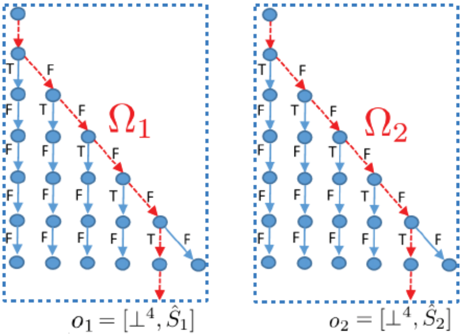

Fig. 11: Two runs of Alg. 2.

Indeed it is enough to apply the sequential composition theorem and get the obvious bound $\frac{\epsilon}{4} \cdot n$.

CRSE can prove this by applying the rule **Proof-Step-Lap-Gen** $n$ times, and then choosing $K_1, \ldots, K_n$ all equal to 0. This would imply the statement of equality of the output variables spending less than $\epsilon$. A potential counterexample can be found in 5 iterations. If we apply strategy B to this algorithm and follow the relational symbolic trace that applies the rule **Proof-Step-Lap-Gen** for all the samplings we can isolate the relational specular trace shown in Figure 11, which corresponds to the left execution following the false branch for the first four iterations and then following the true branch and setting the fifth element of the array to the sampled value. Let's denote the respective final relational configuration by $\langle m_1, m_2, \texttt{skip}, p_1, p_2, s\rangle$. The set of constraints is as follows: $s = \langle \Omega_1, C(\vec{k}), \Omega_2\rangle = \langle\{T_1 > S_1^1, T_1 > S_1^2, T_1 > S_1^3, T_1 > S_1^4, T_1 \leq S_1^5\}, \{T_1 + k_0 = T_2, S_1^1 + k_1 = S_2^1, S_1^2 + k_2 = S_2^2, S_1^3 + k_3 = S_2^3, S_1^4 + k_4 = S_2^4, S_1^5 + k_5 = S_2^5, E_6 = k_0\frac{\epsilon}{2} + \frac{\epsilon}{4}\sum_{i=1}^{4} k_i \dots\}, \{T_2 > S_2^1, T_2 > S_2^2, T_2 > S_2^3, T_2 > S_2^4, T_2 \leq S_2^5\}\rangle$ with $m_1(\epsilon_c) = m_2(\epsilon_c) = E_6, m_1(o) = [S_1^1, \dots, S_1^5], m_2(o) = [S_2^1, \dots, S_2^5], m_1(t) = T_1, m_2(t) = T_2$.

We can see that strategy B applies, because we have $\models \forall\vec{k}.[(\Omega_1 \wedge C(\vec{k}) \implies \Omega_2) \wedge [\![\epsilon_c \leq \epsilon)]\!]_{\langle m_1|m_2\rangle}] \implies [\![o_1 \neq o_2]\!]_{\langle m_1|m_2\rangle}$. Computing the probability associated with these two traces we can verify that we have a counterexample. This pair of traces is, in fact, the same that has been found in [16] for a slightly more general version of Algorithm (2). Strategy B selects this relational trace since in order to make sure that the traces follow the same branches, the coupling rules enforce necessarily that the two samples released are different, preventing CRSE to prove equality of the output variables in the two runs.

**Unsafe sparse vector implementation: Algorithm 3.** Also this algorithm satisfies Assumption 1. The algorithm is $\epsilon$-differentially private for one iteration. This is because, intuitively, adding noise to the threshold protects the result of the query as well at the branching instruction, but only for one iteration. The algorithm is not $\epsilon$-differentially private, for any finite $\epsilon$ already at the second iteration, and a witness for this can be found using CRSE. We can see this using strategy B. Thanks to this strategy we will isolate a relational orthogonal trace, similarly to what has been found in [16] for the same algorithm. CRSE will unfold the loop twice, and it will scan all relational traces to see if there is an orthogonal trace. In particular, the relational trace that corresponds to the output $o_1 = o_2 = [\bot, \top]$, that is the the trace with set of constraints $\langle \Omega_1, C(\vec{k}), \Omega_2\rangle = \langle\{T_1 > q_{1d1}, T_1 \leq q_{2d1}\}, \{|q_{1d1} - q_{1d2}| \leq 1, |q_{2d1} - q_{2d2}| \leq 1\}\{T_2 > q_{1d2}, T_2 \leq q_{2d2}\}\rangle$. Since the vector $\vec{k}$ is empty we can omit it and just write $C$. It is easy to see now that the following sigma: $\sigma \equiv [q_{1d1} \mapsto 0, q_{2d1} \mapsto 1, q_{1d2} \mapsto 1, q_{2d2} \mapsto 0]$, proves that this relational trace is orthogonal: that is $\sigma \models \Omega_1 \wedge C$, but $\sigma \not\models \Omega_2$.

Indeed if we consider two inputs $D_1, D_2$ and two queries $q_1, q_2$ such that: $q_1(D_1) = q_2(D_2) = 0, q_2(D_1) = q_1(D_2) = 1$ we get that the probability of outputting the value $o = [\bot, \top]$ is positive in the first run, but it is 0 on the second. Hence, the algorithm can only be proven to be $\infty$-differentially private.

**A safe sparse vector implementation.** Algorithm 2 can be proven $\epsilon$-d.p if we replace $o[i]\leftarrow\top$ to line 7. Let us consider a proof of this statement for $n = 5$. CRSE will try to prove the following postconditions: $o_1 = [\top, \bot, \dots, \bot] \implies o_2 = [\top, \bot, \dots, \bot] \wedge \epsilon_c \leq \epsilon$, $\dots$, $o_1 = [\bot, \dots, \bot, \top] \implies o_2 = [\bot, \dots, \bot, \top] \wedge \epsilon_c \leq \epsilon$. The only interesting iteration will be the i-th one, in all the others the postcondition will be vacuously true. Also, the budget spent will be $k_0\frac{\epsilon}{2}$, the one spent for the threshold. For all the other sampling instruction we can spend 0 by just setting $k_j = q[j](D_2) - q[j](D_1)$ for $j \neq i$, that is by coupling $\hat{s}_1 + k_j = \hat{s}_2$, with $k_j = q[j](D_2) - q[j](D_1)$, spending $|k_j + q[j](D_2) - q[j](D_1)| = 0$. So, at the i-th iteration the samples are coupled $\hat{s}_1 + k_i = \hat{s}_2$, with $k_i = 1$. So if $\hat{s}_1 \geq \hat{t}_1$ then also $\hat{s}_2 \geq \hat{t}_2$, and also, if $\hat{s}_1 < \hat{t}_1$ then also $\hat{s}_2 < \hat{t}_2$. This implies that at th i-th iteration we enter on the right run the true branch iff we enter the true branch on the left one. This by spending $|k_i + q[i](D_2) - q[i](D_1)|\frac{\epsilon}{4} \leq 2\frac{\epsilon}{4}$. The total privacy budget spent will then be equal to $\epsilon$.

## 9 Related Works

There is now a wide array of formal techniques for reasoning about differential privacy, e.g. [1–6, 12, 15, 18, 20–23, 23, 24, 26, 27]. We will discuss here the techniques that are closest to our work. In [1] the authors devised a synthesis framework to automatically discover proofs of privacy using coupling rules similar to ours. However, their approach is not based on relational symbolic execution but on synthesis technique. Moreover, their framework cannot be directly used to find violations of differential privacy. In [2] the authors devise a decision logic for differential privacy which can soundly prove or disprove differential privacy. The programs considered there do not allow assignments to real and integer variables inside the body of while loops. While their technique is different from our, their logic could be potentially integrated in our framework as a decision procedure. In the recent concurrent work [23], the authors propose an automated technique for proving or finding violations to differential privacy based on program analysis, standard symbolic execution and on the notion of *randomness alignment*, which in their approach plays the role that approximate coupling plays for us here. Their approach focuses on efficiency and scalability, while we focus here more on the fundational aspects of our technique.

Another recent concurrent work [27] combines testing based on (unary) symbolic execution with approximate coupling for proving and finding violations to differential privacy. Their symbolic execution engine is similar to our SPFOR, and is used to reduce the numbers of tests that need to be generated, and for building privacy proofs from concrete executions. Their approach relies more directly on testing, providing an approximate notion of privacy. As discussed in their paper this could be potentially mitigated by using a relational symbolic execution engine as the one we propose here, at the cost of using more complex constraints. Another related work is [15], proposing model checking for finding counterexamples to differential privacy. The main difference with our work is in the basic technique and in the fact that model checking reason about a model of the code, rather than the code itself. They also consider the above threshold example and they are able to handle only a finite number of iterations.

Other work has studied how to find violations to differential privacy through testing [5, 6]. The approaches proposed in [5, 6] differ from ours in two ways: first, they use a statistical approach; second, they look at concrete values of the data and the privacy parameters. By using symbolic execution we are able to reason about symbolic values, and so consider $\epsilon$-differential privacy for any finite $\epsilon$. Moreover, our technique does not need sampling - although we still need to compute distributions to confirm a violation. Our work can be seen as a probabilistic extension of the framework presented in [10], where sampling instructions in the relational symbolic semantics are handled through rules inspired by the logic apRHL$^+$ [3]. This logic can be used to prove differential privacy but does not directly help in finding counterexamples when the program is not private.

### 10 Conclusion

We presented CRSE: a symbolic execution engine framework integrating relational reasoning and probabilistic couplings. The framework allows both proving and refuting differential privacy. When proving CRSE can be seen as strong postcondition calculus. When refuting CRSE uses several strategies to isolate potentially *dangerous* traces. Future work includes interfacing more efficiently CRSE with numeric solvers to find maximums of ratios of probabilities of traces.

*Acknowledgements* We warmly thank the reviewers for helping us improving the paper. This work was supported by the National Science Foundation under Grant No. 1565365, 1565387 and 2040215.
